# Enhanced mTORC1 Signaling in Inflammatory Monocytes Links Systemic Inflammation to Cardiovascular Disease in Rheumatoid Arthritis

**DOI:** 10.3390/biomedicines13112578

**Published:** 2025-10-22

**Authors:** Claudio Karsulovic, Fabian Tempio, Mercedes Lopez, Julia Guerrero, Ka Wei Katty Joo Hu, Annelise Goecke

**Affiliations:** 1 Laboratorio de Inmunomodulación Neuroendocrina, Instituto de Ciencias Biomédicas, Facultad de Medicina, Universidad de Chile, Santiago 8380453, Chile; 2 Seccion de Reumatología, Hospital Clínico Universidad de Chile, Universidad de Chile, Santiago 8380453, Chile; 3 Servicio de Reumatología, Clínica Alemana de Santiago, Universidad del Desarrollo, Santiago 7610315, Chile; 4 Laboratorio de Regulación e Inmunología del Cáncer, Facultad de Medicina, Universidad de Chile, Santiago 8380453, Chile; 5 Instituto Milenio de Inmunología e Inmunoterapia, Facultad de Medicina, Universidad de Chile, Santiago 8380453, Chile; 6 Facultad de Medicina, Universidad del Desarrollo-Clinica Alemana de Santiago, Santiago 7550000, Chile

**Keywords:** rheumatoid arthritis, cardiovascular risk, monocyte subsets, mTOR, IL-1β, IL-6

## Abstract

**Background/Objectives:** Cardiovascular disease (CVD) is the leading cause of mortality in patients with rheumatoid arthritis (RA), not fully explained by traditional risk factors and disease activity alone. This study explored the relationship between circulating monocyte subsets, inflammatory cytokine profiles, and Mammalian Target of Rapamycin Complex (mTORC) signaling in RA patients with and without a history of CVD. **Methods:** Peripheral blood mononuclear cells from 9 RA patients with prior CVD, 9 carefully matched RA controls without CVD, and 6 healthy controls were analyzed by flow cytometry. Matching was rigorously conducted across clinically relevant variables, including age, sex, blood pressure, lipid profile, smoking status, RA duration, disease activity, Disease-Modifying Anti-Rheumatic Drug (DMARD) failures, and steroid use. Monocyte subsets were classified as inflammatory (CD14^+^HLA-DR^+^CCR2^+^) and non-inflammatory (CD14^+^CD163^+^CCR2^−^). **Results:** RA-CVD^+^ patients exhibited higher frequencies of inflammatory monocytes and elevated intracellular levels of Interleukin 1 β (IL-1β) and Interleukin 6 (IL-6) compared to RA-CVD^−^ patients and healthy controls. mTORC activation, assessed by phosphorylation of S6 Ribosomal Protein (S6Rp), was significantly increased in inflammatory monocytes from RA-CVD^+^ patients. **Conclusions:** S6Rp correlated with IL-1β and IL-6 levels only in the RA-CVD^+^ group, suggesting a link between mTORC activity and inflammatory monocyte function. Notably, these inflammatory features did not correlate with disease activity scores or disease duration. We observed increased mTORC1 signaling in inflammatory monocytes in RA-CVD^+^ patients, suggesting a potential association with cardiovascular comorbidity.

## 1. Introduction

Cardiovascular disease (CVD) is the leading cause of excess mortality in rheumatoid arthritis (RA), with myocardial infarction and ischemic stroke being the most frequent events [1]. Even when adjusted for traditional cardiovascular risk factors, RA confers a higher risk of CVD than other chronic conditions such as diabetes [2]. Improving cardiovascular risk prediction and understanding its underlying mechanisms are therefore essential to prevent adverse outcomes and guide personalized interventions.

Traditional tools such as the Framingham Risk Score significantly underestimate cardiovascular risk in RA patients [2]. Adaptations incorporating RA-specific features, including DAS28 values or the 1.5 multiplication factor recommended by EULAR, have only modestly improved predictive accuracy [3,4,5]. This persistent gap highlights the contribution of residual inflammatory mechanisms beyond clinical disease activity [6]. While atherogenesis is a central mechanism, RA patients also experience other cardiovascular outcomes, including ischemic stroke, underscoring the need to investigate immune pathways that bridge systemic inflammation with a spectrum of cardiovascular events [4].

Several regulatory pathways contribute to the excess cardiovascular risk observed in RA. Beyond joint inflammation, activated monocytes and macrophages amplify systemic inflammation through IL-1β, IL-6, TNFα, and CCL2, promoting endothelial dysfunction and vascular remodeling. Canonical signaling cascades such as NF-κB and JAK/STAT drive pro-inflammatory transcriptional programs, while metabolic regulators such as mTORC1 integrate nutrient and inflammatory cues, linking immune activation with vascular damage. This convergence of inflammatory and immunometabolic pathways provides a plausible mechanistic bridge between RA pathogenesis and cardiovascular disease.

Atherogenesis is a complex process that begins early in life and progresses with changes in the arteries’ intima in different territories, leading to its obliteration. This process starts early in the disease in RA patients, even in patients without classic cardiovascular risk factors [7]. Thickness values of the carotid intima-media (IMTc), a recognized cardiovascular risk factor, are elevated in RA patients with low cardiovascular risk estimated by traditional scores [8].

Circulating monocytes, as precursors of tissue macrophages, may contribute to the inflammatory environment that promotes atherogenesis [9,10]. Human peripheral blood monocytes are traditionally categorized into classical (CD14^+^CD16^−^), intermediate (CD14^+^CD16^+^), and non-classical (CD14^low^CD16^+^) subsets. Classical monocytes represent the main inflammatory population, characterized by high CCR2 expression and the ability to extravasate into tissues and secrete pro-inflammatory cytokines in response to stimuli. In contrast, non-classical monocytes patrol the vascular endothelium and exhibit lower cytokine production under homeostatic conditions. This functional heterogeneity highlights the importance of phenotypic characterization when assessing monocyte-mediated inflammation. In post-acute myocardial infarction (AMI) stent re-stenosis, non-inflammatory subtypes of monocyte are augmented [11,12]. In RA, the CD14^+^CD68^+^CCR2^+^ (inflammatory)/CD14^+^CD68^+^CCR2^+^ (non-inflammatory) ratio of circulating monocytes is positively correlated with osteoclast activity [13]. A plausible connector between these observations is the highly conserved adaptation module, Mammalian Target of Rapamycin Complex (mTORC). mTORC inhibitors are widely used to prevent post-AMI stent re-stenosis and, at the same time, are a possible target in modulating macrophage/monocyte-mediated inflammation [14]. In RA patients with previous cardiovascular events, there is no description of monocyte subset behavior, its intracellular inflammatory state, or the activation status of the mTOR complex.

## 2. Materials and Methods

### 2.1. Patients

Recruitment of patients was performed in three stages (Supporting Information Figure S1): First, we identified eleven patients after studying eight-year electronic data by cross-referencing RA with cardiovascular event diagnosis (group 2). After that, we selected our patients who were initially diagnosed with RA and underwent a cardiovascular event, including myocardial infarction or ischemic stroke. Two patients with a systemic sclerosis overlap and diabetes mellitus type 2 diagnoses were excluded. A full rheumatologist consultation was performed, including articular count, DAS28 calculation, lipid profile, and recently obtained inflammatory parameter revision. Blood samples were immediately processed using a mononuclear Ficoll extraction procedure and frozen until analysis. In the second stage, we manually selected twenty RA patients from the same database, identical in all pairing criteria except for the cardiovascular event (all events occurred more than one year before enrollment; group 3). The pairing criteria used were the following: age: ± 5 years; same biological sex; more or less than 140 mmHg of systolic blood pressure in the last outpatient visit; normal or altered High-Density Lipoprotein Cholesterol (according to American Heart Association 2019 guidelines [15]); more or less than 10 Pack-year tobacco smoking; RA time-from-diagnosis ±1 year; more or less than 2 failed DMARDs; Disease Activity Score in 28 Joints using the Erythrocyte Sedimentation Rate (DAS28-ESR): remission, low, moderate, or high disease activity; and steroid use: in use, suspended less than 3 months, 3 months to 6 months, or more than 6 months. The same rheumatologist evaluation confirming the absence of other chronic inflammatory or metabolic diseases was performed before whole-blood extraction. Eleven patients were excluded because of inflammatory or treatment mismatch with our first patients. Nine identical pairs were enrolled. In the third stage, six non-paired healthy controls were included in the analysis using the same clinical and blood extraction protocol (group 1). To ensure comparability, RA patients with and without CVD were carefully matched not only for cardiovascular risk factors but also for clinical RA phenotypes, including disease duration, DAS28 categories, number of DMARD failures, and corticosteroid use. All patients fulfilled ACR/EULAR criteria and were evaluated by the same rheumatologist to confirm homogeneity and representativeness of the cohort. Cardiovascular disease (CVD) was defined as a documented history of myocardial infarction or ischemic stroke, confirmed by clinical records. Patients with other systemic conditions that could confound cardiovascular outcomes, including diabetes mellitus or connective tissue disease overlap, were excluded.

All participants provided informed consent following the Declaration of Helsinki. The protocol was approved by both the Human Research Ethics Committee at Universidad de Chile and the Research Ethics Committee at Hospital Clinico Universidad de Chile (Project Number: 191-2016; Acta Number: 127).

### 2.2. PBMC Extraction and Flow Cytometry

Venous blood (30 mL) was obtained by cubital venipuncture from all participants using 10 mL BD Vacutainer green cap heparin tubes (BD Biosciences, New York, NY, USA). PBMC Ficoll extraction was performed. Cells were washed, followed by staining with the Cell Viability Kit (BD Biosciences, New York, NY, USA) and LUNA cell counter (Brightfield—Logos Biosystems, Gyeonggi-do, Republic of Korea) with a cell viability of 97.4% ± 1.5%. PBMCs were stained in duplicate with the following antibodies at room temperature for 30 min: V710-anti-CD3, V650-anti-CD19, Fluorescein Isothiocyanate (FITC)-A-anti-CD14, Allophycocyanin (APC)-H7-anti CD16, Peridinin-Chlorophyll-Protein (PERCP)-Cy5.5-anti-CD86, APC-A-anti-CD163 (Biolegend, San Diego, CA, USA), V610—Anti Human C-C Chemokine Receptor Type 2 (CCR2) (Biolegend, San Diego, CA, USA), and V785-Anti Human–Human Leukocyte Antigen-DR (HLA-DR) (Biolegend, San Diego, CA, USA). Finally, we fixed and permeabilized cells using a BD Cytofix/Cytoperm fixation/permeabilization kit (BD Biosciences, New York, NY, USA) and stained them with V450-A-anti-Interleukin-1β, Anti Human IL-6 Phycoerythrin (PE) (Biolegend, San Diego, CA, USA), and Phospho-S6 Ribosomal Protein (Ser235/236) Antibody (Cell Signaling Technology, Danvers, MA, USA). The frequency and median fluorescence intensity of monocyte subsets were assessed on a FACS LSRFortessa instrument (BD, Franklin Lakes, NJ, USA). The data were analyzed with FlowJo software (v7.6.1 and v10.7; TreeStar; Ashland, DE, USA).

### 2.3. Gating Strategy

Peripheral blood mononuclear cells (PBMCs) were gated based on forward and side scatter (FSC-A vs. SSC-A), followed by exclusion of doublets and dead cells using a Zombie viability dye. Lineage-positive cells (CD3^+^ T cells, CD19^+^ B cells, CD66b^+^ granulocytes) were excluded through negative gating. Monocytes were defined as CD14^+^ events within the lineage-negative, live population. Two phenotypic subsets were identified: Inflammatory monocytes: CD14^+^HLA-DR^+^CCR2^+^ and non-inflammatory monocytes: CD14^+^CD163^+^CCR2^−^. This approach was based on prior functional profiling of intracellular cytokine production in RA monocytes. While CD14/CD16 subsets are widely used, they do not fully capture inflammatory activation in this context and were not used here. Gating thresholds were set based on fluorescence minus one (FMO) control [16,17].

### 2.4. Statistical Analysis

Statistical analyses were performed using GraphPad Prism v9.0. Sample size was estimated based on anticipated differences in intracellular cytokine expression, informed by prior in vitro studies. Due to the low prevalence of RA patients with well-characterized cardiovascular comorbidity, this approach prioritized biological feasibility over formal power calculation. Normality was assessed using the Shapiro–Wilk test. For comparisons between two groups, unpaired *t*-tests or Mann–Whitney U tests were used depending on distribution. For comparisons among three or more groups, Kruskal–Wallis tests followed by Benjamini–Hochberg false discovery rate (FDR) correction were applied to control for multiple testing. Associations were assessed using Spearman’s rank correlation given the limited sample size and the non-parametric distribution of most variables. Multivariable regression analyses were not feasible in this cohort but represent an important future approach for larger datasets. Spearman correlation coefficients (ρ) were used to assess associations between cytokine-producing monocyte subsets and clinical scores. Only moderate or strong correlations (|ρ| ≥ 0.5) were considered meaningful. Principal Component Analysis (PCA) was applied to visualize multivariate clustering of cytokine and marker expression across monocyte subsets. Variables contributing most to PC1 and PC2 are indicated in the figure legend. Hierarchical clustering was performed using Ward’s method and Euclidean distance after Z-score normalization. PCA was used to identify variance across immunologic and clinical variables. Principal Component Analysis (PCA) and HeatMap analysis were performed using XLSTAT version 2022.2.1 (Addinsoft, New York, NY, USA).

## 3. Results

**Figure 1:**
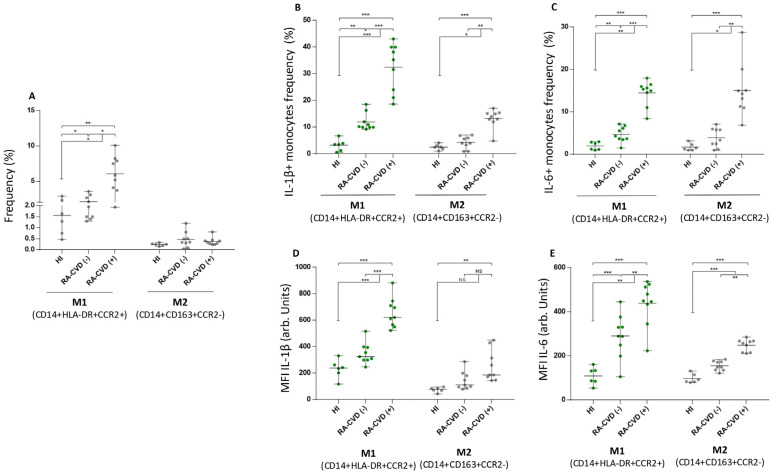
CD14^+^CCR2^+^HLA-DR^+^ and CD14^+^CD163^+^CCR2^−^ monocyte frequency and cytokine signature in cardiovascular and non-cardiovascular events in RA patients. (**A**) Frequency of CD14^+^CCR2^+^HLA-DR^+^ and CD14^+^CD163^+^CCR2^−^ monocytes and intracellular cytokine expression (IL-1β, IL-6) in RA patients with and without cardiovascular events. (**B**) Frequencies of IL-1 β^+^ cells among CD14^+^CCR2^+^HLA-DR^+^ and CD14^+^CD163^+^CCR2^−^ monocytes are presented. Values in healthy subjects and RA CVD-negative and -positive patients are shown. (**C**) Frequency of IL-6^+^ cells among CD14^+^CCR2^+^HLA-DR^+^ and CD14^+^CD163^+^CCR2^−^ monocyte subsets in three study groups. (**D**) MFI levels of IL-1β in CD14^+^CCR2^+^HLA-DR^+^ and CD14^+^CD163^+^CCR2^−^ monocytes from all three groups. (**E**) MFI levels of IL-6 in CD14^+^CCR2^+^HLA-DR^+^ and CD14^+^CD163^+^CCR2^−^ monocytes from the three study groups. Green dots represent M1 monocytes (CD14^+^HLA-DR^+^CCR2^+^), and gray dots represent M2 monocytes (CD14^+^CD163^+^CCR2^−^). Data are shown as mean ± SEM. Significance is indicated as follows: *p* < 0.05 (*), *p* < 0.01 (**), and *p* < 0.001 (***). The Mann–Whitney test was used. HI (Healthy Individual), RA-CVD (Rheumatoid Arthritis with Cardiovascular Disease), IL-1β (Interleukin 1β), IL-6 (Interleukin 6), MFI (Mean Fluorescence Intensity).

As shown in Table 1, nine patients with RA and CVD history were identified with an average of 62 ± 3 years. Five patients were women and eight reported previous myocardial infarctions. In terms of disease activity, the mean of DAS28-ESR was 2.98 ± 0.18, with at least eight of them exhibiting low disease activity. Eight patients were using steroids at the time of evaluation, all of them with doses below 7.5 mg per day. Eight patients failed to less than two DMARDs. DMARD failures were primarily due to lack of efficacy, defined as persistent disease activity after ≥3 months of adequate therapy. Only two patients discontinued a DMARD due to intolerance. At evaluation, 16/18 RA patients were receiving low-dose glucocorticoids (<7.5 mg/day), while two had discontinued corticosteroids more than 6 months prior. The control group’s (RA patients without previous CVE) age, cardiovascular risk factors, and disease activity features showed no statistical difference in the study group. The healthy control group (n = 6) was younger with a mean age of 32.5 ± 7 years and had no disease or medication use (Table 1). Systemic inflammatory markers ESR and CRP were measured at the same visit and are reported in Table 1. Correlation analyses showed no significant association between ESR/CRP and intracellular cytokine expression or mTORC1 activity. Circulating IL-1β and IL-6 in plasma were not available, which we acknowledge as a limitation. Non-inflammatory monocytes in this study refer to CD14^+^CD163^+^CCR2^−^ cells, characterized by lower cytokine expression. Comparisons with healthy controls (HCs) are shown in Figure 1, where intracellular mTORC1 activity was lowest in HC and progressively higher in RA without CVD and RA-CVD^+^ patients. ESR and CRP were measured at the same visit and did not significantly correlate with intracellular cytokines or mTORC1 activity.

We evaluated the frequency of CD14^+^HLA-DR^+^CCR2^+^ and CD14^+^CD163^+^CCR2^−^ in each group. CD14^+^HLA-DR^+^CCR2^+^ but not CD14^+^CD163^+^CCR2^−^ monocytes were higher in RA subjects versus healthy controls (4.08 ± 4.46 vs. 1.55 ± 1.73, *p* < 0.01). Among inflammatory monocytes from RA subjects, those with cardiovascular disease (CVD+) showed a higher frequency in comparison without (CVD^−^) (5.78 ± 4.15 vs. 1.93 ± 1.49, *p* < 0.05) (Figure 1A). Then, IL-1 β^+^ cells were evaluated: RA-CVD^+^ subjects had a higher number of IL-1 β^+^ cells than RA-CVD^−^ patients did (35.1 ± 17.5 vs. 10.3 ± 4.8, *p* < 0.01) (Figure 1B). CD14^+^CD163^+^CCR2^−^ monocytes also showed a higher frequency of IL-1 β^+^ cells in RA-CVD^+^ subjects. Similar results were found for median fluorescence intensity (MFI) values (622 ± 162 vs. 325 ± 95, *p* < 0.001) (Figure 1D). The same analysis was performed for frequency and MFI of IL-6, showing that in CD14^+^HLA-DR^+^CCR2^+^ and CD14^+^CD163+CCR2^−^, RA-CVD^+^ subjects have expanded inflammatory subsets versus RA-CVD^−^ and healthy controls (Figure 1C,E).

**Figure 2:**
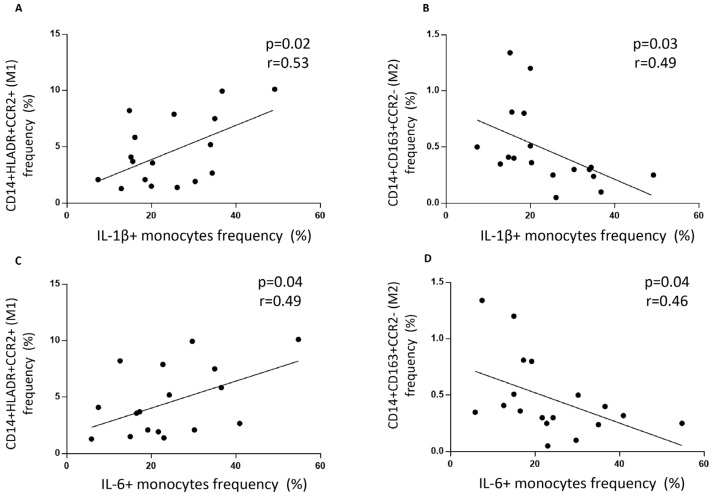
Monocyte phenotype correlation with intracellular cytokine levels in rheumatoid arthritis patients. (**A**) Correlation between CD14^+^CCR2^+^HLA-DR^+^ monocyte frequency and IL-1 β^+^ cells among CD14^+^ total monocytes. (**B**) Correlation between CD14^+^CD163^+^CCR2^−^ monocyte frequency and IL-1 β^+^ cells among CD14^+^ total monocytes. (**C**) Correlation between CD14^+^CCR2^+^HLA-DR^+^ monocyte frequency and IL-6^+^ cells among CD14^+^ total monocytes. (**D**) Correlation between CD14^+^CD163^+^CCR2^−^ monocyte frequency and IL-6^+^ cells among CD14^+^ total monocytes. The Spearman rank correlation test was used.

To determine each monocyte subset with its inflammatory or non-inflammatory cytokine mark, we correlated the frequency of CD14^+^HLA-DR^+^CCR2^+^ monocytes with their intracellular levels of IL-1β and IL-6 cytokines. CD14^+^HLA-DR^+^CCR2^+^ monocytes correlated positively with intracellular levels of IL-1β (r = 0.53, *p* < 0.05) and IL-6 (r = 0.49, *p* < 0.05) (Figure 2A,C), while CD14^+^CD163^+^CCR2^−^ monocytes inversely correlated with IL-1β (r = −0.49, *p* < 0.05) and IL-6 (r = −0.46, *p* < 0.05) (Figure 2B,D).

**Figure 3:**
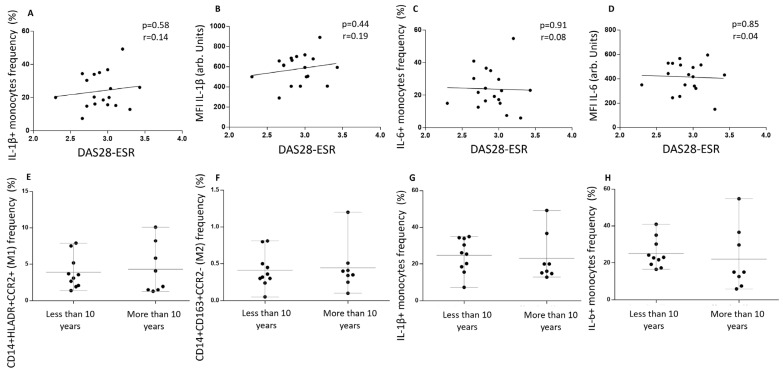
DAS28 and disease duration and their correlation with inflammatory cytokines and monocyte subsets. (**A**) Correlation between DAS28-ESR and frequency of IL-1β^+^ cells among CD14^+^ total monocytes. (**B**) Correlation between DAS28-ESR and IL-1β MFI in CD14^+^ total monocytes. (**C**) Correlation between DAS28-ESR and frequency of IL-6^+^ cells among CD14^+^ total monocytes. (**D**) Correlation between DAS28-ESR and IL-6 MFI in CD14^+^ total monocytes. (**E**) Frequency of CD14^+^HLA-DR^+^CCR2^+^ monocytes in RA patients with more/equal or less than 10 years of the disease. (**F**) Frequency of CD14^+^CD163^+^CCR2^−^ monocytes in RA patients with more/equal or less than 10 years of the disease. (**G**) Frequency of IL-1 β^+^ cells among CD14^+^ total monocytes in RA patients with more/equal or less than 10 years of the disease. (**H**) Frequency of IL-6^+^ cells among CD14^+^ total monocytes in RA patients with more/equal or less than 10 years of the disease. Mann–Whitney and Spearman rank correlation tests were used.

To determine whether disease activity, measured using DAS28 at the time the blood sample was taken, associated with cytokine values and monocyte phenotype, we analyzed and correlated these values. IL-1 β^+^ frequency and MFI did not correlate with DAS28-ESR levels (Figure 3A,B). The same results were found when IL-6 values were analyzed (Figure 3C,D).

Since disease duration has been described as a prognostic factor, we divided our cohort into RA patients with more or less than ten years from diagnosis. The frequencies of CD14^+^HLA-DR^+^CCR2^+^ and CD14^+^CD163^+^CCR2^−^ monocytes were the same in both groups (Figure 3E,F). Also, the number of IL-1 β^+^ and IL-6^+^ cells was the same among patients with more or less than ten years of RA diagnosis, showing that clinical features did not correlate with monocyte phenotypes or intracellular inflammatory cytokines in this setting (Figure 3G,H).

**Figure 4:**
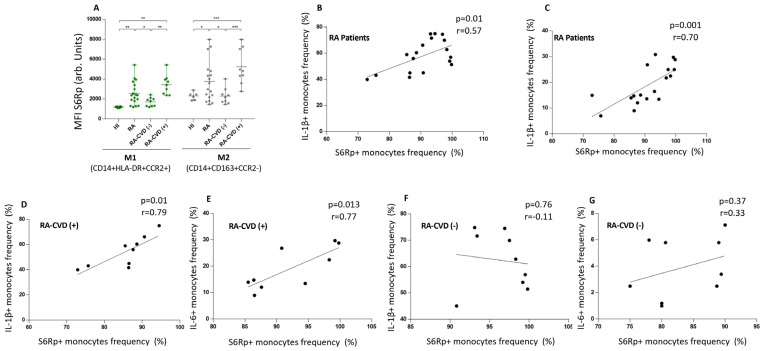
Intracellular mTORC activity analysis in RA patients with and without cardiovascular disease. (**A**) Intracellular S6R phosphorylated levels in CD14^+^HLA-DR^+^CCR2^+^ and CD14^+^CD163^+^CCR2^−^ monocytes from healthy subjects, total RA, and RA-CVD-negative and -positive patients. (**B**) Correlation between IL-1 β^+^ cells and S6Rp^+^ cells among CD14^+^ total monocytes. (**C**) Correlation between IL-6^+^ cells and S6Rp+ cells among CD14^+^ total monocytes. (**D**) Correlation between IL-1 β^+^ cells and S6Rp+ cells among CD14^+^ total monocytes from RA patients with a previous CVD event. (**E**) Correlation between IL-6^+^ cells and S6Rp+ cells among CD14^+^ total monocytes from RA patients with a previous CVD event. (**F**) Correlation between IL-1 β^+^ cells and S6Rp+ cells among CD14^+^ total monocytes from RA patients without a previous CVD event. (**G**) Correlation between IL-6^+^ cells and S6Rp+ cells among CD14^+^ total monocytes from RA patients without a previous CVD event. (**A**) Data are shown as mean ± SEM. Significance is indicated as follows: *p* < 0.05 (*), *p* < 0.01 (**), *p* < 0.001 (***); (**B**) r = 0.57 (**C**) r = 0.70, (**D**) r = 0.79, (**E**) r = 0.77, (**F**) r = −0.11 (ns), (**G**) r = 0.33 (ns). Mann–Whitney and Spearman rank correlation tests were used.

mTOR activity, measured as the phosphorylation of S6R protein, showed higher intracellular levels in RA subjects than healthy controls (2613 ± 1290 vs. 1200 ± 180, *p* < 0.001). Among them, inflammatory monocytes from RA-CVD^+^ patients had the highest values (3414 ± 1691) (Figure 4A). Then, we analyzed the cytokine profile and the S6Rp levels. RA subjects showed that IL-1 β^+^ and IL-6^+^ cell frequency positively correlated with S6Rp intracellular levels (r = 0.57, *p* < 0.01 and r = 0.70, *p* < 0.001) (Figure 4B,C). When separated into RA-CVD^+^ and RA-CVD^−^ patients, both IL-1 β^+^ and IL-6^+^ cells positively correlated with S6Rp levels in CVD^+^ (r = 0.79, *p* < 0.01 and r = 0.77, *p* < 0.01) (Figure 4D,E) but not in RA-CVD- subjects (Figure 4F,G).

**Figure 5:**
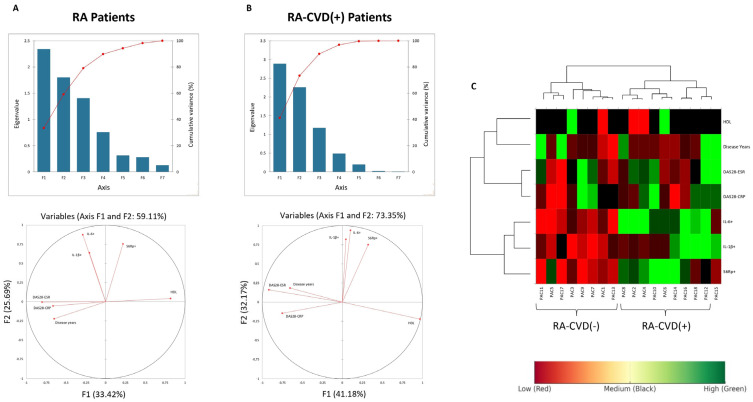
Dimensional reduction of clinical, cytokine, and monocyte phenotype features in RA patients with and without CVD. (**A**,**B**) Variables’ PC1 vs. PC2 plot. PC1 + PC2 accounts for 59.11% of variable power in the non-CVD event subgroup and 73.35% in the previous CVD patient group. PC1 reflects variance in disease activity (DAS28-ESR and disease duration) and PC2 is primarily associated with IL-1β^+^, IL-6^+^, and pS6^+^ monocytes. (**C**) Heatmap shows featured clinical values, frequency of inflammatory cytokine-positive cells, and mTOR protein values in all patients. RA-CVD-negative and RA-CVD-positive patients group together. Definitions: Principal Component 1 (PC1), Principal Component 2 (PC2), cardiovascular disease (CVD), Rheumatoid Arthritis with/without Cardiovascular Disease (RA-CVD), Mammalian Target of Rapamycin Complex (mTORC), High-Density Lipoprotein (HDL), Disease Activity Score in 28 Joints using the Erythrocyte Sedimentation Rate (DAS28-ESR), Disease Activity Score in 28 Joints using C-Reactive Protein (DAS-CRP), Interleukin-1 beta (IL-1b), Interleukin-6 (IL-6), S6 Ribosomal Protein (S6Rp). Clustering was performed using Ward’s method and Euclidean distance after Z-score normalization.

To better assess these differences, we added a mathematical approach analyzing all variables at once to establish clearer correlations. Principal Component Analysis was performed to explore patterns of variance across immunologic and clinical variables. Variables with larger loadings on PC1 or PC2 contributed more to the explained variance captured by those components. Cytokines such as IL-1β and IL-6 exhibited strong loadings on PC2, whereas disease activity measures such as DAS28-ESR were primarily associated with PC1, suggesting that they represent distinct, orthogonal dimensions of variation in RA patients (Figure 5A,B). The lack of clustering between cytokines and clinical scores further supports their independent behavior in this dataset. Given their previously observed correlation with S6R phosphorylation, this axis of variation may reflect an immunometabolic pathway relevant to cardiovascular risk in RA (Figure 5C).

## 4. Discussion

Cardiovascular disease remains the leading cause of mortality in patients with RA, with up to 50% experiencing a CVD event during the disease course. Even when adjusted for traditional cardiovascular risk factors [4], RA confers a higher risk of CVD than other chronic conditions such as diabetes. Improving cardiovascular risk prediction and understanding its underlying mechanisms are essential to prevent adverse outcomes and guide personalized interventions.

Traditional tools such as the Framingham Risk Score significantly underestimate CVD risk in RA patients. Attempts to incorporate disease-specific features into these models have only marginally improved predictive accuracy [18]. As a pragmatic workaround, current EULAR guidelines recommend applying a 1.5 multiplication factor to the Framingham score in RA [19]. However, growing evidence suggests that low disease activity values in DAS28 alone does not eliminate CVD risk, pointing to additional drivers beyond clinical inflammation [20].

Circulating monocytes have emerged as key mediators of vascular inflammation and atherogenesis in chronic inflammatory conditions. In both murine and human models of atherosclerosis, CD14^+^CD16^−^CCR2^+^ monocytes are expanded and exhibit increased trafficking to atheromatous plaques [21,22]. Recent studies suggest this same inflammatory subset is also expanded in RA patients with CVD [23].

Building on these observations, we analyzed monocyte phenotypes in RA patients with and without prior CVD, using a carefully matched case–control design. We found that RA patients with prior CVD had a significantly higher frequency of CCR2^+^HLA-DR^+^ inflammatory monocytes. These cells also showed increased expression of IL-1β and IL-6, two key cytokines linked to inflammasome and NF-κB activation pathways, respectively.

Interestingly, cytokine expression did not correlate with clinical disease activity scores such as DAS28-CRP or ESR. Despite close matching for disease activity and traditional CV risk factors, monocyte inflammation remained elevated in patients with CVD, suggesting the presence of residual inflammatory risk not captured by standard clinical indices supporting the concept of residual subclinical inflammation not captured by DAS28 or ESR. Biomarkers like IL-6 or TNFα, or modalities such as ultrasound, may provide more sensitive assessments of vascular inflammation in RA.

Our findings support the existence of an immunometabolic signature in RA patients with cardiovascular comorbidity, characterized by CCR2^+^HLA-DR^+^ monocytes with enhanced IL-1β/IL-6 production and increased mTORC1 activity. This profile may serve as a candidate biomarker for residual cardiovascular risk, complementing clinical scores such as DAS28. Beyond risk stratification, targeting immunometabolic nodes such as mTORC1 could represent a novel therapeutic approach to reduce cardiovascular burden in RA.

We selected mTORC1 as the primary pathway of interest because, unlike NF-κB and JAK/STAT that regulate transcriptional programs, mTORC1 integrates inflammatory and metabolic signals at the translational level. This allows mTORC1 to directly influence cell growth, cytokine production, and vascular remodeling. Thus, mTORC1 complements canonical pathways by acting as an immunometabolic hub that amplifies residual cardiovascular risk in RA.

**Figure 6:**
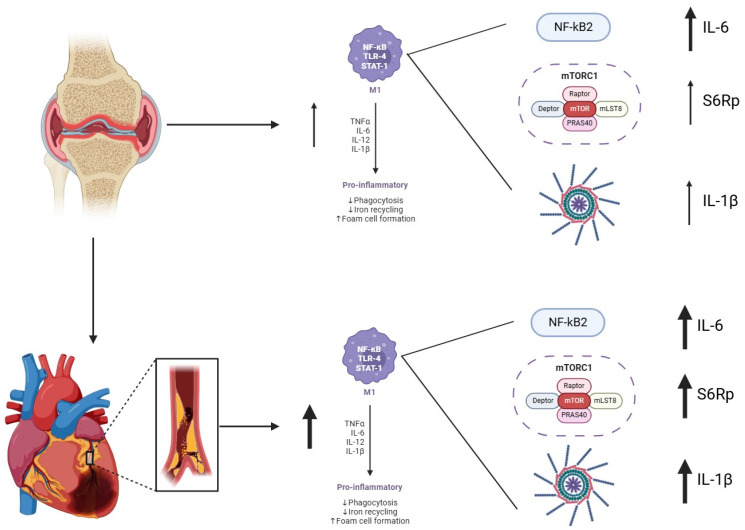
Schematic representation of the link between rheumatoid arthritis (RA) and cardiovascular disease (CVD) through enhanced inflammatory signaling in monocytes. In RA, synovial inflammation induces M1 monocytes with increased pro-inflammatory cytokines and functional alterations. Inflammatory monocytes from RA-CVD^+^ patients exhibit enhanced NF-κB2 and mTORC1-S6Rp signaling, with increased IL-6 and IL-1β expression, linking systemic inflammation to vascular damage.

In this context, we explored the activation of the mTORC, a key metabolic regulator implicated in immune cell proliferation, trafficking, and plaque progression. Our findings showed an increased phosphorylation of S6Rp, a downstream mTORC effector, in RA patients with CVD. Notably, S6Rp levels correlated with inflammatory cytokines only in CCR2^+^ monocytes, suggesting a subset-specific amplification of inflammation via mTORC signaling. This aligns with previous data showing altered mTOR activity in RA synovial tissue and highlights a potential circulating correlation of tissue inflammation [24]. Although our findings support an association between mTORC activation and cardiovascular comorbidity in RA, the directionality of this relationship remains uncertain. mTORC may reflect a downstream effect of systemic inflammation rather than a direct cause (Figure 6). Additionally, increased mTOR signaling has been observed in other inflammatory conditions with cardiovascular risk, suggesting it is not specific to RA [25].

The functional classification of circulating monocytes in humans remains limited, as their phenotype often reflects transient activation states rather than stable polarization profiles. Unlike tissue macrophages, circulating monocytes lack a definitive in vivo classification. In our study, we used a marker-based approach (CD14^+^CCR2^+^HLA-DR^+^) to define an inflammatory phenotype, based on prior evidence of their relevance in monocyte migration and activation [26]. This strategy, while operational, acknowledges the current limitations in resolving monocyte function solely through peripheral surface markers [27].

Our study was strengthened by the rigorous clinical matching across cardiovascular and rheumatologic variables. Nevertheless, its retrospective design, small sample size, and exploratory nature are limitations. Our sample size was based on expected cytokine differences from prior in vitro work, which limits generalizability but reflects the feasibility constraints of studying rare RA-CVD^+^ cases. Prospective studies in larger cohorts are needed to evaluate whether CCR2^+^ monocyte activation and S6Rp expression represent reliable biomarkers or therapeutic targets. If validated, these immunometabolic pathways may open the door for precision cardiovascular risk stratification and the use of mTOR-targeted interventions in high-risk RA patients.

We focused on IL-1β and IL-6 because of their central roles in inflammasome activation and mTORC1 signaling. Other key cytokines such as TNFα and CCL2 were not measured in this study, which we acknowledge as a limitation. Importantly, all immunological determinations were performed simultaneously with the clinical evaluation and DAS28 scoring, ensuring that cytokine and signaling data reflect the same disease activity time point.

All IL-1β and IL-6 measurements reported correspond to basal intracellular levels assessed ex vivo without stimulation. As expected, variability was greater in inflammatory (CD14^+^HLA-DR^+^CCR2^+^) than in non-inflammatory (CD14^+^CD163^+^CCR2^−^) subsets. Vascular biomarkers such as VCAM-1 or ICAM-1 were not measured in this cohort, which we recognize as a limitation for interpreting vascular involvement.

Anti-inflammatory cytokines such as IL-10 and TGF-β were not measured in this study. We acknowledge this as a limitation and note that assessing the balance between pro- and anti-inflammatory mediators may provide further insight into residual cardiovascular risk in RA.

Future studies should evaluate larger RA cohorts with longitudinal follow-up to establish the predictive value of CCR2^+^ monocyte activation and mTORC1 signaling for cardiovascular events. Interventional studies targeting mTOR pathways may help clarify causality and therapeutic potential. In addition, integrating vascular biomarkers and anti-inflammatory cytokines would provide a more comprehensive view of residual cardiovascular risk in RA. Finally, a limitation of our study is that systemic cytokines IL-1β and IL-6 were not assessed in plasma; however, ESR and CRP were measured at the same visit and did not significantly correlate with intracellular cytokine or mTORC1 activity.

## Figures and Tables

**Table 1:** Demographic, clinical, and cardiovascular risk factors in RA patients with and without cardiovascular events. Values are presented as mean ± SD for continuous variables and n (%) for categorical variables. Groups were matched for age, sex, RA disease duration, DAS28-CRP, and cardiovascular risk profile. RA-CVD^+^: RA patients with prior cardiovascular event; RA-CVD^−^: RA patients without cardiovascular event. NS: not significant.

	Group 1 Healthy (N = 6)	Group 2 RA-CVD^+^ (N = 9)	Group 3 RA-CVD^−^ (N = 9)	*p*-Value G2 vs. G3
**Demographic**				
Age, *years ± SD*	32.5 ± 7	62 ± 3	63 ± 2	*NS*
Men, *n*	3	4	4	
Female, *n*	3	5	5	
**RA Characteristics**				
Diagnosis, *years* ± *SD*		11 ± 5	11 ± 6	*NS*
DAS28-RCP, *score* ± *SD*		2.96 ± 0.23	2.88 ± 0.43	*NS*
DAS28-ESR, score ± SD		2.98 ± 0.18	2.97 ± 0.30	*NS*
Systemic steroids, *n*		9	9	
In use		8	8	
Suspended +6 months		1	1	
DMARDS Failure, *n*		9	9	
<2 DMARDS		8	8	
>2 DMARDS		1	1	
bDARMDS in use, *n*		2	2	
**CVD Characteristics**				
Myocardial Infarction, *n*	0	8	0	
Stroke, *n*	0	1	0	
OPAD, *n*	0	0	0	
**Cardiovascular Risk Factors**				
HTN, *n*	0	8	8	
Lipid Disorder, *n*	0	7	7	
HDL, *mg*/*dL* ± SD	45.2 ± 7.2	35.50 ± 4.89	38.86 ± 4.47	*NS*
Tobacco use, (*Pack-year*) ± SD	0	7 ± 12.25	2.57 ± 6.8	*NS*
**Medication**				
Statins, *n*	0	9	7	*NS*

## Data Availability

All data generated or analyzed during this study are included in this published article and Supplementary Information. Raw flow cytometry data (FCS files) and FlowJo workspaces used in this study are available from the corresponding author upon reasonable request.

## Supplementary Materials

The following supporting information can be downloaded at https://www.mdpi.com/article/10.3390/biomedicines13112578/s1. Figure S1: Flowchart of patient and control selection. The study was conducted in three stages. In Stage 1, RA patients with a confirmed cardiovascular (CV) event during treatment were identified; two were excluded due to systemic sclerosis overlap and diabetes mellitus type 2, resulting in nine RA-CVD^+^ patients. In Stage 2, matched RA patients without CV events were selected based on clinical and laboratory data; eleven preselected candidates were excluded due to mismatch in DAS28 interval, steroid misuse, or undocumented treatment changes, resulting in nine matched RA-CVD^−^ patients. In Stage 3, six healthy controls were recruited and underwent the same clinical and laboratory evaluation. All participants signed informed consent and provided blood samples for immunological analysis.

## Abbreviations

The following abbreviations are used in this manuscript:

AOSD	Adult-Onset Still’s Disease
ANAs	Antinuclear Antibodies
CRP	C-Reactive Protein
DMARDs	Disease-Modifying Anti-Rheumatic Drugs
ESR	Erythrocyte Sedimentation Rate
HIV	Human Immunodeficiency Virus
ICAM-1	Intercellular Adhesion Molecule-1
IL	Interleukin
JIA	Juvenile Idiopathic Arthritis
MAS	Macrophage Activation Syndrome
MCP	Metacarpophalangeal
MTX	Methotrexate
NF-κB	Nuclear Factor kappa B
PET-CT	Positron Emission Tomography–Computed Tomography
sCD25	Soluble Interleukin-2 Receptor alpha
VEGF	Vascular Endothelial Growth Factor

## References

[1] Humphreys J.H., Warner A., Chipping J., Marshall T., Lunt M., Symmons D.P.M., Verstappen S.M.M. (2014). Mortality trends in patients with early rheumatoid arthritis over 20 years: Results from the Norfolk Arthritis Register. Arthritis Care Res..

[2] Fryar C.D., Chen T.C., Li X. (2012). Prevalence of Uncontrolled Risk Factors for Cardiovascular Disease: United States, 1999–2010.

[3] Arts E.E., Fransen J., den Broeder A.A., Popa C.D., van Riel P.L. (2015). The effect of disease duration and disease activity on the risk of cardiovascular disease in rheumatoid arthritis patients. Ann. Rheum. Dis..

[4] Popescu D., Rezus E., Badescu M.C., Dima N., Isac P.N.S., Dragoi I.-T., Rezus C. (2023). Cardiovascular risk assessment in rheumatoid arthritis: Accelerated atherosclerosis, new biomarkers, and the effects of biological therapy. Life.

[5] Arts E.E., Popa C.D., Den Broeder A.A., Donders R., Sandoo A., Toms T., Rollefstad S., Ikdahl E., Semb A.G., Kitas G. (2016). Prediction of cardiovascular risk in rheumatoid arthritis: Performance of original and adapted SCORE algorithms. Ann. Rheum. Dis..

[6] Peters M.J., Symmons D.P., McCarey D., Dijkmans B.A.C., Nicola P., Kvien T.K., McInnes I.B., Haentzschel H., Gonzalez-Gay M.A., Provan S. (2010). EULAR evidence-based recommendations for cardiovascular risk management in patients with rheumatoid arthritis and other forms of inflammatory arthritis. Ann. Rheum. Dis..

[7] Saliba-Gustafsson P., Pedrelli M., Gertow K., Werngren O., Janas V., Pourteymour S., Baldassarre D., Tremoli E., Veglia F., Rauramaa R. (2019). Subclinical atherosclerosis and its progression are modulated by PLIN2 through a feed-forward loop between LXR and autophagy. J. Intern. Med..

[8] Ikdahl E., Rollefstad S., Wibetoe G., Olsen I.C., Berg I.-J., Hisdal J., Uhlig T., Haugeberg G., Kvien T.K., Provan S.A. (2016). Predictive value of arterial stiffness and subclinical carotid atherosclerosis for cardiovascular disease in patients with rheumatoid arthritis. J. Rheumatol..

[9] Lu D., Jiao X., Jiang W., Yang L., Gong Q., Wang X., Wei M., Gong S. (2023). Mesenchymal stem cells influence monocyte/macrophage phenotype: Regulatory mode and potential clinical applications. Biomed. Pharmacother..

[10] Colin S., Chinetti-Gbaguidi G., Staels B. (2014). Macrophage phenotypes in atherosclerosis. Immunol. Rev..

[11] Moroni F., Ammirati E., Norata G.D., Magnoni M., Camici P.G. (2019). The role of monocytes and macrophages in human atherosclerosis, plaque neoangiogenesis, and atherothrombosis. Mediat. Inflamm..

[12] SahBandar I.N., Ndhlovu L.C., Saiki K., Kohorn L.B., Peterson M.M., D’Antoni M.L., Shiramizu B., Shikuma C.M., Chow D.C. (2020). Relationship between circulating inflammatory monocytes and cardiovascular disease measures of carotid intimal thickness. J. Atheroscler. Thromb..

[13] Rana A.K., Li Y., Dang Q., Yang F. (2018). Monocytes in rheumatoid arthritis: Circulating precursors of macrophages and osteoclasts and, their heterogeneity and plasticity role in RA pathogenesis. Int. Immunopharmacol..

[14] Chen C., Wang J., Liu C., Hu J., Liu L. (2023). Pioneering therapies for post-infarction angiogenesis: Insight into molecular mechanisms and preclinical studies. Biomed. Pharmacother..

[15] Grundy S.M., Stone N.J., Bailey A.L., Beam C., Birtcher K.K., Blumenthal R.S., Braun L.T., De Ferranti S., Faiella-Tommasino J., Forman D.E. (2019). 2018 AHA/ACC/AACVPR/AAPA/ABC/ACPM/ADA/AGS/APhA/ASPC/NLA/PCNA Guideline on the management of blood cholesterol: A report of the American college of cardiology/american heart association task force on clinical practice guidelines. Circulation.

[16] Thomas G.D., Hamers A.A.J., Nakao C., Marcovecchio P., Taylor A.M., McSkimming C., Nguyen A.T., McNamara C.A., Hedrick C.C. (2017). Human blood monocyte subsets: A new gating strategy defined using cell surface markers identified by mass cytometry. Arterioscler. Thromb. Vasc. Biol..

[17] Karsulovic C., Tempio F., Lopez M., Guerrero J., Goecke A. (2021). In vitro phenotype induction of circulating monocytes: CD16 and CD163 analysis. J. Inflamm. Res..

[18] Dessein P.H., Stanwix A.E., Solomon A. (2020). Could disease activity score in 28 joints-gamma-glutamyl transferase use improve cardiovascular disease risk management in rheumatoid arthritis?. J. Rheumatol..

[19] Agca R., Heslinga S.C., Rollefstad S., Heslinga M., McInnes I.B., Peters M.J., Kvien T.K., Dougados M., Radner H., Atzeni F. (2017). EULAR recommendations for cardiovascular disease risk management in patients with rheumatoid arthritis and other forms of inflammatory joint disorders: 2015/2016 update. Ann. Rheum. Dis..

[20] Charles-Schoeman C., Buch M.H., Dougados M., Bhatt D.L., Giles J.T., Ytterberg S.R., Koch G.G., Vranic I., Wu J., Wang C. (2023). Risk of major adverse cardiovascular events with tofacitinib versus tumour necrosis factor inhibitors in patients with rheumatoid arthritis with or without a history of atherosclerotic cardiovascular disease: A post hoc analysis from ORAL Surveillance. Ann. Rheum. Dis..

[21] Julla J.B., Girard D., Diedisheim M., Saulnier P.J., Tran Vuong B., Bleriot C., Carcarino E., De Keizer J., Orliaguet L., Nemazanyy I. (2024). Blood monocyte phenotype is a marker of cardiovascular risk in type 2 diabetes. Circ. Res..

[22] Verweij S.L., Duivenvoorden R., Stiekema L.C.A., Nurmohamed N.S., van der Valk F.M., Versloot M., Verberne H.J., Stroes E.S.G., Nahrendorf M., Bekkering S. (2018). CCR2 expression on circulating monocytes is associated with arterial wall inflammation assessed by 18F-FDG PET/CT in patients at risk for cardiovascular disease. Cardiovasc. Res..

[23] Rose S., Eren M., Murphy S., Zhang H., Thaxton C.S., Chowaniec J., Waters E.A., Meade T.J., Vaughan D.E., Perlman H. (2013). A novel mouse model that develops spontaneous arthritis and is predisposed towards atherosclerosis. Ann. Rheum. Dis..

[24] Barker B.E., Hanlon M.M., Marzaioli V., Smith C.M., Cunningham C.C., Fletcher J.M., Veale D.J., Fearon U., Canavan M. (2023). The mammalian target of rapamycin contributes to synovial fibroblast pathogenicity in rheumatoid arthritis. Front. Med..

[25] Joo K., Karsulovic C., Sore M., Hojman L. (2024). Pivotal Role of mTOR in Non-Skin Manifestations of Psoriasis. Int. J. Mol. Sci..

[26] Loyola K., Karsulovic C., Cabrera R., Perez C., Hojman L. (2023). New markers for cardiovascular disease in psoriatic patients: Preliminary study on monocyte phenotype, ADAMTS7, and mTOR activity. Metabolites.

[27] Blériot C., Chakarov S., Ginhoux F. (2020). Determinants of Resident Tissue Macrophage Identity and Function. Immunity.

[28] Goecke A., Karsulovic C., Guerrero J., Tempio F., Lopez M. (2020). SAT0004 Increased M1 Inflammatory phenotype of circulating monocytes is associated with history of cardiovascular events in RA Patients. Ann. Rheum. Diseases..

