# Supplementary material for: Enhanced mTORC1 Signaling in Inflammatory Monocytes Links Systemic Inflammation to Cardiovascular Disease in Rheumatoid Arthritis
Source: Biomedicines. 2025 Oct 22;13(11):2578. doi: 10.3390/biomedicines13112578 (PMC12649968; doi:10.3390/biomedicines13112578)
Supplement: Supplementary file 1 [file biomedicines-13-02578-s001.zip › biomedicines-3851008-supplementary.pdf]

biomedicines

MDPI

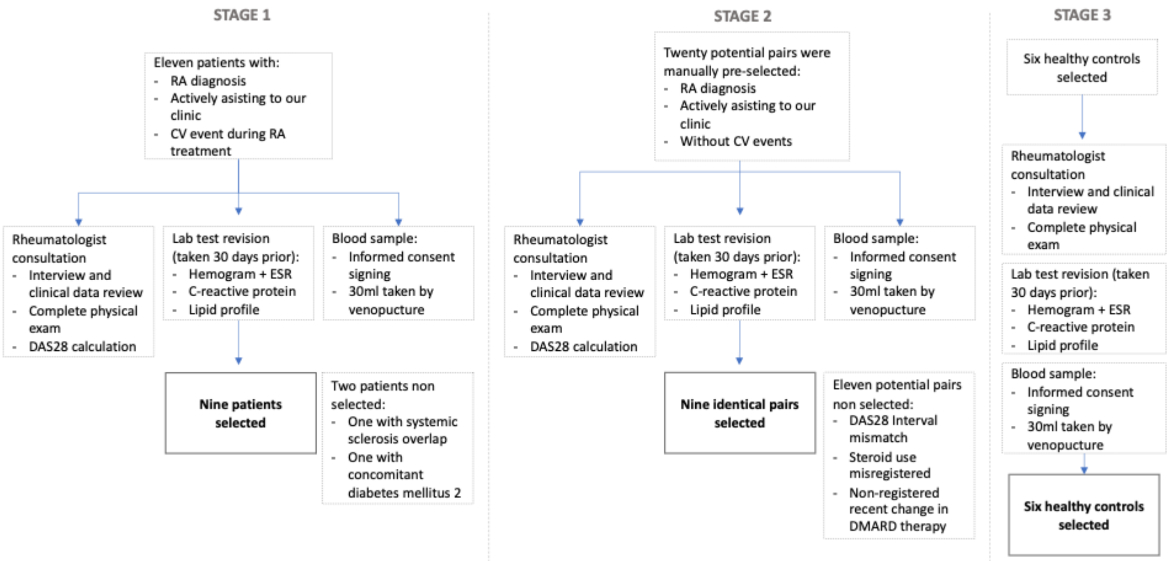

Figure S1. Flowchart of patient and control selection. The study was conducted in three stages. In Stage 1, RA patients with a confirmed cardiovascular (CV) event during treatment were identified; two were excluded due to systemic sclerosis overlap and diabetes mellitus type 2, resulting in nine RA-CVD$^+$ patients. In Stage 2, matched RA patients without CV events were selected based on clinical and laboratory data; eleven preselected candidates were excluded due to mismatch in DAS28 interval, steroid misuse, or undocumented treatment changes, resulting in nine matched RA-CVD$^-$ patients. In Stage 3, six healthy controls were recruited and underwent the same clinical and laboratory evaluation. All participants signed informed consent and provided blood samples for immunological analysis.
